# Molecular Advances in Juvenile Myelomonocytic Leukemia and Associated RASopathy

**DOI:** 10.3390/cancers18101655

**Published:** 2026-05-20

**Authors:** Fnu Monika, Sara Abu Mehsen, Ling Zhang

**Affiliations:** 1 Department of Pathology, H. Lee Moffitt Cancer Center & Research Institute, Tampa, FL 33612, USAsara.abumehsen@moffitt.org (S.A.M.); 2 Morsani College of Medicine, University of South Florida, Tampa, FL 33612, USA

**Keywords:** JMML, MPN, RASopathy, five canonical driver genes (*PTPN11*, *NRAS*, *KRAS*, *NF1*, and *CBL*), DNA methylation, Noonan syndrome, CBL syndrome, neurofibromatosis type 1 (NF1), RAS-MAPK pathway

## Abstract

Juvenile myelomonocytic leukemia (JMML) is a rare, aggressive myeloproliferative neoplasm of early childhood characterized by constitutive activation of the RAS-MAPK signaling pathway. RASopathies are a heterogeneous group of complex genetic disorders arising from germline mutations that dysregulate RAS-MAPK signaling. Noonan syndrome, CBL syndrome, and neurofibromatosis type 1 (NF1) are the three major RASopathies predisposing to JMML. More than 90% of JMML cases harbor germline or somatic mutations in one of five canonical driver genes—*PTPN11*, *NRAS*, *KRAS*, *NF1*, or *CBL*—establishing JMML as the prototypical malignant manifestation of RASopathy biology. The fifth edition of the World Health Organization Classification of Tumours reclassified JMML as a myeloproliferative neoplasm while the International Consensus Classification adopted JMML under pediatric and/or germline mutation-associated disorders, introducing a JMML-like category for cases lacking five canonical mutations but harboring emerging drivers such as *SH2B3::LNK* alterations and *ALK::ROS1* fusions. The distinction between germline and somatic mutations profoundly influences prognosis: e.g., germline *PTPN11*-associated myeloproliferations and many germline *CBL* cases undergo spontaneous resolution, whereas somatic *PTPN11-* and *NF1*-mutated JMML is more aggressive and requires prompt allogeneic hematopoietic stem cell transplantation. DNA methylation profiling has emerged as the most robust prognostic framework, with consensus defining high-, intermediate-, and low-methylation subgroups that independently predict outcome. Both genotype and DNA methylation subclassification have been integrated into clinical decision-making, incorporating pretransplant azacitidine, watch-and-wait approaches for favorable-risk patients, and emerging targeted therapies including MEK inhibitors. This review synthesizes recent advances in understanding JMML as a bona fide RASopathy; provides a diagnostic algorithm, molecular landscapes, and prognostic models; and highlights opportunities for molecularly targeted therapeutic intervention.

## Simple Summary

Juvenile myelomonocytic leukemia (JMML) is a rare, aggressive myeloproliferative neoplasm of early childhood characterized by constitutive RAS-MAPK pathway activation. More than 90% of cases harbor mutations in one of five canonical driver genes—*PTPN11*, *NRAS*, *KRAS*, *NF1*, or *CBL.* Approximately 25–30% of cases arise in children with germline RASopathy-associated mutations, while the majority (55–65%) are driven by somatically acquired mutations in the same pathway genes; this shared convergence on RAS-MAPK hyperactivation has led to the conceptualization of JMML as a bona fide RASopathy. Clinical outcomes vary dramatically based on mutation origin: e.g., somatic *PTPN11* mutations confer poor prognosis, requiring prompt hematopoietic stem cell transplantation, while germline *CBL* and select *NRAS* mutations may undergo spontaneous resolution with watchful waiting. DNA methylation profiling has emerged as the most robust prognostic tool that independently predicts survival. In this review, we aim to (1) update current concepts of JMML and its relationship to RASopathies, (2) discuss RASopathy-associated genetic variants and their roles in RAS-MAPK signaling, and (3) explore potential targetable and emerging inhibitors of the RAS-MAPK pathway.

## 1. Introduction

Juvenile myelomonocytic leukemia (JMML) is a rare, aggressive myeloproliferative neoplasm (MPN) of infancy and early childhood, with an estimated incidence of approximately 1.2 per million children per year and a median age at diagnosis of 2 years (range, 0–14 years) [1,2]. Juvenile myelomonocytic leukemia accounts for approximately 1% of all pediatric leukemias and 20% to 40% of pediatric myelodysplastic syndromes (MDS), with a male predominance (male-to-female ratio, approximately 2:1) [3]. More than 90% of cases harbor mutations in one of five canonical RAS-pathway genes—*PTPN11*, *NRAS*, *KRAS*, *NF1*, or *CBL* [4,5]. Two-thirds of those cases occur as sporadic somatic mutations, and one-third arise among children with germline RASopathy-associated predisposition that requires additional somatic events for leukemic transformation [4,6].

Clinically, children present with constitutional symptoms, marked hepatosplenomegaly (80–90%), lymphadenopathy, and cutaneous manifestations [7,8,9]. Leukemic infiltration of the lungs is a recognized complication that may cause respiratory symptoms, whereas CNS involvement is rare [7,8,9]. Laboratory evaluation characteristically reveals leukocytosis with absolute monocytosis (≥1 × 10^9^/L), anemia, thrombocytopenia, and elevated fetal hemoglobin (HbF) for age [7,10,11]. Bone marrow examination demonstrates hypercellularity with myelomonocytic proliferation; blasts and promonocytes are characteristically not expanded and together must comprise less than 20% of nucleated cells to distinguish JMML from acute leukemia [7]. While the majority of patients require allogeneic hematopoietic stem cell transplantation (HSCT) for long-term survival, a subset of cases undergoes spontaneous disease resolution, making molecular subtyping essential for risk stratification and treatment planning [11,12].

## 2. Evolution of JMML Classification

The classification of JMML has evolved over the past two decades, reflecting advances in understanding its distinct molecular pathogenesis as a RAS-driven malignancy (Table 1). Juvenile myelomonocytic leukemia was initially classified in the myelodysplastic/myeloproliferative neoplasm (MDS/MPN) category in the World Health Organization Classification of Tumours of Haematopoietic and Lymphoid Neoplasms3rd (WHO-3; 2001) and 4th (WHO-4; 2008) editions, reflecting its combined dysplastic and proliferative features [13,14]. The revised WHO-4 (2016) updated diagnostic criteria by integrating accumulating clinical, morphologic, immunophenotypic, and genetic insights [15]; by this time, approximately 90% of cases were recognized to harbor alterations in one of five canonical RAS-pathway genes, following the landmark identification of *PTPN11* mutations in JMML in 2003 [4,15,16,17]. Notably, the 2016 criteria allowed a diagnosis of JMML based on either a clonal cytogenetic abnormality or molecular evidence of RAS-pathway activation [15]. The fifth edition of the WHO Classification of Tumours (WHO-5) reclassified JMML as an MPN, based on its predominantly proliferative phenotype, near-universal RAS-MAPK pathway mutations, virtual absence of morphologic dysplasia, and clinical manifestation of leukocytosis and organomegaly, which are characteristic of myeloproliferation [18]. However, this reclassification has been critically discussed within the field [19]. Notably, the concurrently published International Consensus Classification (ICC)-2022 proposed a distinct framework by classifying JMML within the category of “pediatric and/or germline mutation-associated disorders” rather than strictly as an MPN, emphasizing its pediatric specificity and frequent association with inherited RASopathies [20,21]. The divergence between these two classification systems reflects ongoing debate about whether JMML’s biology is best captured by its proliferative phenotype (favoring MPN classification) or by its unique pediatric presentation and germline RASopathy associations (favoring a distinct category). This lack of consensus underscores the need for continued refinement of JMML classification as molecular understanding evolves. Both updated classifications place substantially greater emphasis on molecular diagnostics compared with WHO-4. Furthermore, the WHO-5 now requires absence of *KMT2A* rearrangement as a diagnostic criterion for JMML, recognizing that *KMT2A*-rearranged AML can mimic JMML at presentation [18,22]. Additionally, cases presenting with isolated monosomy 7 in the absence of canonical RAS-pathway mutations should not be classified as JMML but rather prompt evaluation for pediatric MDS or germline predisposition syndromes (such as *SAMD9*, *SAMD9L*, and *GATA2*) [23,24]. Importantly, the ICC introduced an additional JMML-like category to encompass cases presenting with clinical and morphologic features of JMML but lacking identifiable canonical RAS-pathway mutations [20,21]. This distinction is clinically significant, as approximately 5% to 10% of clinically diagnosed JMML cases do not harbor mutations in any one of the five canonical driver genes [4,25]. Recent molecular studies have identified novel genetic drivers in these mutation-negative cases, including germline bi-allelic *SH2B3/LNK* mutations affecting Janus Kinase and Signal Transducer and Activator of Transcription (JAK-STAT) signaling and recurrent tyrosine kinase fusions involving *ALK*, *ROS1*, *PDGFRB*, *FLT3*, and *PDGFRA*—the latter particularly reported among older patients with JMML [18,26,27,28]. These discoveries carry therapeutic implications: *SH2B3*-mutated cases may be candidates for *JAK* inhibitors, while *ALK* fusion-positive cases have demonstrated responses to crizotinib, a tyrosine kinase inhibitor specific to ALK1, as well as ROS1 or c-Met [27,28]. The JMML-like category thus serves as a provisional classification acknowledging the clinical similarity of these cases to JMML while recognizing their distinct molecular pathogenesis and the need for continued characterization.

## 3. Pathogenesis and Molecular Biology

**Figure 1:**
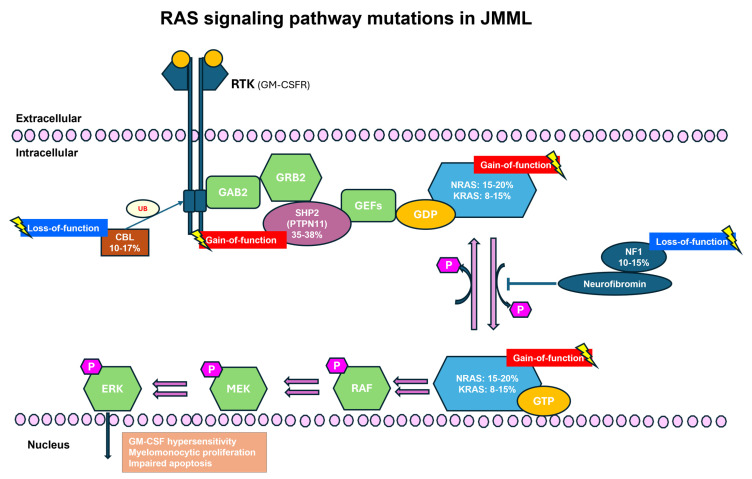
RAS-MAPK signaling pathway and JMML-associated mutations. Activation of Receptor Tyrosine Kinases (RTKs), e.g., GM-CSF receptor (GM-CSFR), initiates signaling through adaptor proteins (GRB2 and GAB2) and the SHP2 phosphatase (encoded by *PTPN11*). Guanine nucleotide exchange factors promote RAS activation by facilitating GDP-to-GTP exchange. Active RAS-GTP promotes the MAPK cascade (RAF→MEK→ERK), driving nuclear transcription and cellular proliferation. Neurofibromin (encoded by *NF1*) normally inactivates RAS by promoting GTP hydrolysis, while CBL ubiquitinates activated RTK receptors for degradation. In JMML, mutations in 5 canonical genes (*PTPN11*, *NRAS*, *KRAS*, *NF1*, and *CBL*), indicated by lightning bolt symbols, converge on pathway hyperactivation: **gain-of-function** mutations in *PTPN11* (35–38%), *NRAS* (15–20%), and *KRAS* (8–15%) enhance signaling, while **loss-of-function** mutations in *NF1* (10–15%) and *CBL* (10–17%) remove negative regulatory checkpoints. The functional consequences include GM-CSF hypersensitivity, abnormal myelomonocytic proliferation, and impaired apoptosis. Abbreviations: ERK, extracellular signal-regulated kinase; GAB2, GRB2-associated binding protein 2; GDP, guanosine diphosphate; GEF, guanine nucleotide exchange factor; GM-CSF, granulocyte–macrophage colony-stimulating factor; GM-CSFR, GM-CSF receptor; GRB2, growth factor receptor-bound protein 2; GTP, guanosine triphosphate; MEK, mitogen-activated protein kinase; P, phosphorylation; RAF, rapidly accelerated fibrosarcoma kinase; RTK, receptor tyrosine kinase; SHP2, Src homology region 2-containing protein tyrosine phosphatase 2.

The pathogenesis of JMML is fundamentally rooted in constitutive activation of the RAS-MAPK signaling pathway (Figure 1), which drives the characteristic myelomonocytic proliferation and hypersensitivity to granulocyte–macrophage colony-stimulating factor (GM-CSF) that define this disease [4,11]. The canonical driver genes—*PTPN11*, *NRAS*, *KRAS*, *NF1*, and *CBL*—converge on hyperactivation of RAS signaling through distinct mechanisms [4,5]. *PTPN11* encodes the protein tyrosine phosphatase SHP-2, and gain-of-function mutations lead to sustained RAS-Guanosine Triphosphate (RAS-GTP) levels and prolonged Extracellular signal-Regulated Kinase (ERK) activation following cytokine stimulation [29]. Similarly, activating mutations in *NRAS* and *KRAS* lock RAS proteins in their GTP-bound active state, while loss-of-function mutations in *NF1* eliminate neurofibromin’s GTPase-activating function, thereby impairing RAS-GTP hydrolysis [30,31]. *CBL* mutations disrupt E3 ubiquitin ligase activity, leading to elevated JAK2- and LYN-kinase expression and enhanced downstream signaling through both RAS-MAPK and Phosphoinositide 3-Kinase-AKT (PI3K-AKT) pathways [32]. The functional consequence of these diverse genetic lesions is a common phenotype: granulocyte–macrophage colony-stimulating factor (GM-CSF) hypersensitivity of myeloid progenitors, which spontaneously forms colonies and enhances proliferation at subthreshold cytokine concentrations [29,30].

Beyond the initiating RAS-pathway mutations, disease progression is driven by secondary genetic and epigenetic alterations. Recurrent secondary mutations in *SETBP1*, *JAK3*, and components of the Polycomb repressive complex 2 (PRC2), including *ASXL1*, contribute to clonal evolution and are associated with inferior outcomes [5,6,33]. The detailed prognostic implications of these secondary mutations are discussed below in the section "Secondary Mutations". Notably, RAS-pathway hyperactivation has been associated with an aberrant DNA methylation through upregulation of the DNA methyltransferases *DNMT1* and *DNMT3B*, providing the biological basis for methylation-based prognostic models that have transformed JMML risk stratification [16] (discussed below). This integrated understanding of the genomic and epigenomic landscape has transformed JMML risk stratification and provides a foundation for developing molecularly targeted therapeutic strategies.

## 4. RASopathy

RASopathies comprise a diverse group of complex, multisystem developmental disorders, caused by germline mutations in genes’ encoding components or modulators of the RAS-MAPK signaling cascade, leading to dysregulated pathway activation [34,35]. The historical evolution of RASopathies reflects a shift from recognizing isolated clinical syndromes to understanding a unified group of developmental disorders. This group of disorders is caused by germline dysregulation of the RAS-MAPK signaling pathway, with major advances in molecular characterization over the past three decades [35,36,37]. Early entities such as Noonan syndrome (NS) and neurofibromatosis type 1 (NF1) were described decades before their molecular basis was understood, initially defined by shared phenotypic features, including facial dysmorphism, cardiac abnormalities, short stature, and developmental delay [35,36]. The molecular era began in the 1990s with the identification of *NF1* [38,39,40] and other disease-associated genes [41] leading to the introduction of the term RASopathies, because these syndromes were unified by shared pathway dysregulation [36]. As gene discovery progressed, the RASopathy spectrum expanded to include NF1, NS, NS with multiple lentigines (NS-ML), cardiofaciocutaneous (CFC) syndrome, Costello syndrome, Legius syndrome, central conducting lymphatic anomalies syndrome, SYNGAP1 syndrome, and capillary malformation arteriovenous malformation syndrome, all caused by pathogenic variants affecting components or regulators of the RAS-MAPK cascade [34,35]. To date, more than 20 RASopathy-associated genes have been identified, with multiple mechanisms of pathway hyperactivation, including increased RAS-GTPase activity, enhanced upstream signaling, dysregulated MAPK effectors, and impaired negative feedback [37]. Collectively, RASopathies affect approximately 1 in 1000 newborns and are now recognized as disorders that are clinically overlapping but genetically heterogeneous [5,25,42]. Importantly, this work established a biological link between germline RAS-pathway dysregulation and hematologic disease. Juvenile myelomonocytic leukemia emerged as the prototypical hematologic malignancy associated with RASopathies, with syndromic cases arising from germline RASopathy-associated mutations (*NF1*, *CBL*, and *PTPN11*) and sporadic cases driven by somatically acquired mutations (*PTPN11*, *NRAS*, and *KRAS*) [25,43,44,45]. Children with RASopathies, particularly the syndromes of NF1, NS, and CBL, have an increased risk of developing JMML or JMML-like myeloproliferative disorder (MPD)—a term used in the RASopathy literature to denote transient, often self-limiting myeloproliferative processes that clinically mimic JMML but are distinct from the traditional WHO classification of MPN [45,46,47] highlighting the continuum between developmental RAS-signaling dysregulation and malignant myeloproliferation [5,44]. The major RASopathy syndromes recognized as predisposing to JMML development are discussed below.

### 4.1. Noonan Syndrome

Noonan syndrome represents one of the most prevalent genetic developmental disorders, affecting approximately 1 in 1000 to 2500 live births, and serves as the prototypical RASopathy [48,49]. First clinically characterized over 50 years ago, NS is now understood to result from germline mutations in genes that encode components of the RAS-MAPK signaling pathway. Heterozygous gain-of-function mutations in *PTPN11* (encoding the protein tyrosine phosphatase SHP-2) are the most common cause, identified in accounting for approximately 50% of cases [49,50]. Mutations in genes including *SOS1*, *RAF1*, *RIT1*, *KRAS*, *NRAS*, *BRAF*, *SHOC2*, and *CBL* account for a further 25% to 30%, and the remaining 20% to 25% of cases lack identifiable molecular etiology [51,52]. Noonan syndrome follows an autosomal-dominant inheritance with complete penetrance but with variable expression, with approximately half of cases arising de novo and the remainder inherited from an affected parent [50]. Clinically, NS manifests with distinctive craniofacial features that evolve with age, including hypertelorism, ptosis, low-set posteriorly rotated ears, webbed neck, short stature, chest deformities (superior pectus carinatum with inferior pectus excavatum), and variable cognitive impairment [48,53]. Cardiovascular involvement occurs among approximately 80% of affected individuals, with pulmonary valve stenosis (50–60%) and hypertrophic cardiomyopathy (16–25%) being the most characteristic cardiac manifestations [47,48,54].

The *PTPN11* gene, located on chromosome 12q24.1, encodes the nonreceptor protein tyrosine phosphatase SHP-2 and is the most commonly mutated gene in NS [46,50]. Most pathogenic variants are missense mutations, resulting in single amino acid substitutions [51]. In addition, pathogenic variants cluster predominantly in exons 3, 8, and 13, which encode regions critical for SHP-2 autoinhibition and catalytic regulation [50,52]. The most commonly identified pathogenic variants occur in exon 8 (c.922A>G and c.923A>G, encoding p.Asn308Asp and p.Asn308Ser), observed among approximately 20% of mutation-positive patients [52]. These mutations disrupt the interaction between the N-SH2 and phosphatase domains, favoring an open enzymatically active conformation and resulting in gain-of-function signaling with enhanced MAPK-pathway activation [46,50]. Notably, mutation location influences JMML risk: N-SH2 domain mutations (exon 3), including those at codons 61, 71, 72, and 76 (Table 2), confer greater JMML risk than PTP domain or exon 8 variants, while the common p.Asn308Asp/Ser (exon 8) carries lower JMML risk [48,49]. The p.Glu76Lys substitution is predominantly a somatic mutation in JMML, while rarely presents as a germline variant. This germline variant causes severe neonatal-lethal NS rather than NS-MPD [46,48,49].

Importantly, children with NS are predisposed to hematologic abnormalities, most notably a transient myeloproliferative disorder (NS-MPD) that clinically resembles JMML [45,47]. Noonan syndrome–MPD is typically diagnosed in the neonatal period or early infancy, with an estimated 10% of these cases progressing to overt JMML [47,55]. Within a large prospective cohort of 641 patients with germline *PTPN11* mutations, myeloproliferative features were identified in 5.6% of patients, with 3% fully meeting diagnostic criteria for JMML [45]; approximately 21% of patients with codon Asp61 mutations developed MPD/JMML in infancy, whereas those with p.Thr73Ile showed a milder, often self-resolving course [45,46]. Critically, the germline *PTPN11* variants associated with NS-MPD differ from the somatic *PTPN11* mutations found in sporadic JMML, which have been functionally demonstrated to confer stronger gain-of-function activity [46,56,57,58,59]; the clinical implications of this distinction, including divergent prognosis and management, are discussed in detail in the section *PTPN11*-Mutated JMML below.

### 4.2. Neurofibromatosis Type 1

Neurofibromatosis type 1, the first RASopathy to be recognized, is the most common inherited disorder of the nervous system and the second most prevalent RASopathy after NS [34]. Neurofibromatosis type 1 is an autosomal-dominant disorder caused by pathogenic variants in the *NF1* tumor suppressor gene on chromosome 17q11.2, with an estimated incidence of approximately 1 in 2500 to 3500 live births [34,50,60]. Neurofibromatosis type 1 is often classified as a RASopathy because of its involvement of the RAS-MAPK signaling pathway; phenotypically it manifests with neurofibromas, café au lait macules, and optic pathway gliomas [61,62]. *NF1* gene encodes neurofibromin, a GTPase-activating protein that negatively regulates RAS signaling [63]. The association between NF1 and JMML has been recognized for several decades with a markedly increased risk of developing JMML, estimated at 200- to 500-fold higher than in the general population [5,64,65]. Approximately 10% to 15% of JMML cases occur among children with NF1, making it the most common inherited cancer-predisposition syndrome associated with this leukemia [61,66]. A subset of NF1-associated JMML cases has also been reported in conjunction with juvenile xanthogranuloma, although the magnitude and independence of this risk remain debated [67]. Clinically, children with NF1-associated JMML present at an older age, with higher bone marrow blast percentages and relatively preserved platelet counts compared with other JMML subtypes [7]. The molecular mechanisms of leukemogenesis and treatment approaches are discussed in detail below in the section *NF1*-Mutated JMML.

### 4.3. CBL Syndrome

CBL syndrome, formally designated as an NS-like disorder with or without JMML, is a rare RASopathy caused by heterozygous germline mutations in the *CBL* gene on chromosome 11q23 [68,69]. The *CBL* gene encodes an E3 ubiquitin ligase that negatively regulates receptor tyrosine kinase signaling; pathogenic mutations affecting the RING finger and linker domains (exons 8–9) result in sustained RAS-MAPK and PI3K-AKT pathway activation [68,70]. CBL syndrome follows autosomal-dominant inheritance with variable expressivity and incomplete penetrance [71,72]. Clinically, CBL syndrome presents with a variable phenotype that overlaps with but is distinct from classic NS. Characteristic features include developmental delay and neurological abnormalities (which occur at relatively high frequency), facial dysmorphism, impaired growth, and cryptorchidism in males [48,71]. Notably, cardiac defects are less prevalent compared with classic NS [47,71]. Additional reported features include café au lait macules (22%), juvenile xanthogranuloma (16%), thin hair (10%), lymphedema, and fetal hydrops or chylothorax [73,74]. Some individuals develop vasculitis later in life, and immune dysregulation with an autoimmune lymphoproliferative syndrome-like phenotype has been described in certain families [69,75].

The most clinically significant feature of CBL syndrome is predisposition to JMML. Among patients with germline CBL mutations, a substantial proportion develops JMML in infancy or early childhood, although the exact penetrance remains incompletely defined [69,76]. The molecular mechanisms of leukemogenesis and clinical management are discussed below in Section 5.5.

Besides the aforementioned three major subtypes, NS, NF1, and CBL syndrome, the following several other RASopathies are also being increasingly linked to JMML-like myeloproliferation.

### 4.4. Costello Syndrome

Costello syndrome (CS) is a rare RASopathy caused exclusively by heterozygous germline *HRAS* mutations, most commonly affecting codons 12 (p.Gly12Ser, p.Gly12Ala, p.Gly12Cys) and 13 (p.Gly13Cys), which result in constitutive activation of the RAS-MAPK pathway [77,78]. Clinically, it is characterized by failure to thrive, developmental delay, distinctive facial and cutaneous features, and significant cardiac involvement, and is associated with an increased lifetime risk of malignancy [78,79,80]. Recent reports have described children with Costello-like phenotypes who developed JMML-like myeloproliferation, highlighting that germline RAS-pathway hyperactivation even outside the canonical JMML driver genes may predispose to myelomonocytic proliferation [81].

While HRAS mutations are not currently considered canonical JMML drivers [18,82], this likely reflects intrinsic biological differences between RAS isoforms—including distinct membrane localization and signaling outputs—rather than rarity alone, as experimental studies demonstrate isoform-specific differences in leukemogenic potential [83,84,85]. Nonetheless, emerging reports of JMML-like myeloproliferation in CS and the ICC recognition of noncanonical RAS-pathway variants suggest the spectrum of JMML-associated drivers may be broader than currently defined.

### 4.5. Cardiofaciocutaneous Syndrome

Cardiofaciocutaneous (CFC) syndrome is a rare RASopathy caused by germline mutations in *BRAF* (most common), *MAP2K1*, *MAP2K2*, or *KRAS*, resulting in constitutive RAS-MAPK pathway activation [86,87]. Clinically, CFC syndrome shares phenotypic overlap with NS and CS, featuring characteristic craniofacial dysmorphism, congenital heart defects, ectodermal abnormalities, and neurocognitive impairment [86]. Although malignancy risk in CFC syndrome is considered lower than in other RASopathies, a single case report has documented transient MDS/MPN in an infant with a germline BRAF c.721A>C mutation, which resolved spontaneously without treatment [88]. Additionally, monocytosis has been observed among approximately 32% of patients with CFC syndrome in recent cohort studies [89]. While overt JMML has not been definitively reported in CFC syndrome, these observations suggest that hematologic surveillance may be warranted.

### 4.6. NRAS-Associated RASopathy

Germline *NRAS* mutations represent a rare cause of RASopathy, with fewer than 30 cases reported in the literature [90]. Affected individuals exhibit phenotypes within the NS spectrum, including short stature, cardiac defects, and facial dysmorphism [90]. Importantly, unlike somatic *NRAS* mutations in cancer, germline variants were initially thought to spare the oncogenic hotspots (codons 12, 13, and 61); however, recent reports have identified germline Gly12 mutations associated with MPDs [90]. One patient with a germline *NRAS* p.Gly12Asp mutation developed an MPD, and another with *NRAS* p.Gly12Arg exhibited a brain tumor, suggesting that mutations at traditionally oncogenic codons confer increased malignancy risk [90,91]. These findings expand the genotype–phenotype spectrum of *NRAS*-associated RASopathy and highlight the need for cancer surveillance in affected individuals.

The estimated JMML/MPD risk, clinical course, and distinguishing features across the major RASopathy syndromes are summarized in Table 3.

## 5. Molecular Subtypes of JMML

Juvenile myelomonocytic leukemia is characterized by mutually exclusive driver mutations in one of five canonical RAS-pathway genes, each defining a distinct molecular subtype with characteristic clinical features, prognosis, and treatment implications (Table 4). These subtypes—*PTPN11*, *NRAS*, *KRAS*, *NF1*, and *CBL—*differ not only in mutation frequency but also in the germline vs. somatic origin of the driver mutation, methylation profile, and clinical behavior. The following sections detail the molecular mechanisms, clinical characteristics, and management considerations for each subtype.

### 5.1. PTPN11-Mutated JMML

Somatic *PTPN11* mutations represent the most common molecular subtype of JMML, accounting for approximately 35% to 38% of all cases [17,94]. The *PTPN11* gene encodes the nonreceptor protein tyrosine phosphatase SHP-2, which functions as a positive regulator of RAS-MAPK signaling downstream of growth factor and cytokine receptors [17,56]. Leukemia-associated *PTPN11* mutations cluster predominantly in exon 3 (encoding the N-SH2 domain) and exon 13 (encoding the PTP domain), with the Glu76Lys substitution being the most frequently observed alteration [17,46]. These mutations disrupt the autoinhibitory interaction between the N-SH2 and PTP domains, resulting in constitutive phosphatase activation and sustained RAS-pathway signaling [56]. Functionally, *PTPN11*-mutated hematopoietic progenitor cells exhibit characteristic GM-CSF hypersensitivity, aberrant myeloid differentiation, and enhanced replating capacity in colony-forming assays [56]. Critically, the spectrum of somatic *PTPN11* mutations in sporadic JMML differs from the germline variants observed in NS. Functional studies have demonstrated that leukemia-associated somatic *PTPN11* mutations confer stronger gain-of-function effects than NS-associated germline variants, with greater capacity for bone marrow transformation and more potent SHP-2 catalytic activation [56,59,95]. Consistent with this, global knock-in of the JMML-associated *PTPN11* E76K mutation causes early embryonic lethality in mice [58], and the rare identification of these variants as germline mutations in humans has been associated with severe neonatal-lethal forms of NS [57].

The clinical behavior of *PTPN11*-mutated JMML differs dramatically based on mutation origin. Somatic *PTPN11* mutations are associated with older age at presentation—1.7 years (range 0.1–9.8 years)—elevated HbF levels, and significantly poorer outcomes compared with other molecular subtypes [96]. *PTPN11*-mutated cases cluster within the high methylation (HM) subgroup, associated with inferior outcomes [16] (see Section 10). Given this unfavorable prognosis, prompt allogeneic HSCT is recommended for all children with somatic *PTPN11*-mutated JMML, and pretransplant therapy with azacitidine may provide clinical benefit [11,97].

In contrast, JMML occurring in the context of NS (germline *PTPN11* mutations) runs a more benign course, with most cases resolving spontaneously without requiring HSCT [47,55,98]. However, this favorable outcome is not consistent across all patients, as severe neonatal presentations are associated with high early mortality, up to 60% in some series, and JMML remains the major cause of death for individuals with *PTPN11*-mutated NS [45]. Residual monocytosis and splenomegaly may persist for several years even after clinical improvement, and long-term surveillance remains essential given the persistence of clonal hematopoiesis and the eight-fold-increased overall risk of childhood malignancy in NS [47,55,99]. Distinguishing germline mutations from somatic *PTPN11* mutations through constitutional tissue testing is therefore essential for prognostication and treatment planning.

### 5.2. NRAS-Mutated JMML

Somatic mutations in NRAS occur in approximately 15% to 20% of JMML cases, making it the second most common molecular subtype among the sporadic forms of the disease [4,94]. *NRAS* encodes a small GTPase that cycles between active GTP-bound and inactive GDP-bound states, serving as a critical node in the RAS-MAPK signaling cascade [4]. Oncogenic *NRAS* mutations, typically affecting codons 12, 13, or 61, impair intrinsic GTPase activity and render the protein constitutively active, resulting in sustained MEK-ERK signaling, GM-CSF hypersensitivity, and uncontrolled myelomonocytic proliferation [30]. Unlike *PTPN11-*mutated JMML, the *NRAS*-mutated subtype demonstrates considerable clinical heterogeneity, with outcomes ranging from spontaneous resolution to aggressive disease requiring transplantation [97,98]. *NRAS*-mutated cases are enriched in the low methylation (LM) subgroup, correlating with favorable prognosis [16] (see the below Secition 10. Specific clinical features predictive of favorable outcomes in *NRAS*-mutated JMML include (1) age less than 30 months at diagnosis, (2) normal to mildly elevated HbF, (3) platelet count above 45 × 10^9^/L, (4) absence of secondary somatic mutations, and (5) low DNA methylation profile [92]. Consequently, a watch-and-wait approach may be appropriate for selected *NRAS*-mutated patients who meet these favorable criteria, potentially sparing them from transplant-associated morbidity [92]. However, patients with *NRAS* mutations who exhibit HM profiles or harbor additional genetic alterations should be considered for allogeneic HSCT, as these features portend more aggressive disease behavior [97].

### 5.3. KRAS-Mutated JMML

Somatic *KRAS* mutations account for approximately 8% to 15% of JMML cases and represent a molecularly and clinically distinct subtype [4,94]. Like *NRAS*, *KRAS* encodes a small GTPase that functions as a molecular switch in the RAS-MAPK pathway, and oncogenic mutations at codons 12, 13, or 61 result in constitutive activation of downstream signaling [30]. However, *KRAS*-mutated JMML exhibits unique biological and clinical characteristics that distinguish it from its *NRAS*-mutated counterpart. Despite shared effector-binding regions, KRAS and NRAS differ in membrane localization, post-translational modifications, and leukemogenic potential, which in JMML manifest as distinct epigenetic and cytogenetic profiles [83,100]. *KRAS*-mutated cases cluster within the intermediate methylation (IM) subgroup (see Section 10) and show a notable association with monosomy 7 [16]. Despite these concerning features, emerging evidence suggests that *KRAS*-mutated JMML may respond favorably to hypomethylating agent (HMA) therapy. Recent clinical experience indicates that upfront azacitidine can achieve durable long-term remissions in some *KRAS*-mutated patients without the need for HSCT [101,102]. The distinct therapeutic responsiveness of *KRAS*-mutated JMML underscores the importance of molecular subtyping in guiding treatment decisions and highlights the potential for mutation-specific therapeutic strategies.

### 5.4. NF1-Mutated JMML

*NF1*-mutated JMML represents a paradigmatic example of inactivation of tumor suppressor genes in myeloid leukemogenesis [4,66]. Unlike the heterozygous gain-of-function mutations in *PTPN11*, *NRAS*, and *KRAS*, *NF1*-mutated JMML requires bi-allelic inactivation of the *NF1* gene for leukemic transformation [64]. Children with neurofibromatosis type 1 inherit a germline loss-of-function mutation in one *NF1* allele; the somatic second hit that inactivates the remaining wild-type allele occurs through several distinct mechanisms: uniparental disomy (UPD) of chromosome 17q via mitotic recombination (approximately 40% of cases), compound heterozygous mutations (approximately 36%), or microdeletions combined with point mutations (approximately 20%) [66,103,104]. The complete loss of neurofibromin function results in constitutive activation of RAS-MAPK and PI3K-AKT pathways, driving GM-CSF hypersensitivity, excessive myelomonocytic proliferation, and resistance to apoptosis—hallmark features of JMML—which have been recapitulated in murine models with hematopoietic-specific *NF1* deficiency [63,105,106].

Importantly, *NF1*-driven JMML can also occur in children without clinical stigmata of neurofibromatosis. For example, in one study, 50% of the patients with *NF1*-mutated JMML lacked obvious NF1 features at diagnosis, underscoring the importance of comprehensive *NF1* analysis in the genetic workup of all JMML cases that lack mutations in the other four canonical driver genes [66,107]. From a molecular standpoint, *NF1*-mutated JMML frequently harbors secondary mutations in genes such as *ASXL1* and *SETBP1*, which contribute to disease progression and are associated with inferior outcomes [66]. Clinical data further suggests that *FLT3* mutations in patients with *NF1* loss of heterozygosity contribute to disease progression [108].

Clinically, *NF1*-mutated JMML behaves aggressively and is uniformly fatal without successful allogeneic HSCT [11,97]. *NF1*-mutated cases show variable methylation profiles but are often enriched in IM to HM subgroups [16] (see Section 10). Current treatment recommendations include pretransplant therapy with azacitidine to reduce disease burden, followed by HSCT with early discontinuation of post-transplant immunosuppression to maximize graft-versus-leukemia effects [97]. Despite these measures, relapse remains a significant challenge, occurring in approximately 50% of transplanted patients, highlighting the urgent need for potential novel targeted therapeutic strategies [106].

### 5.5. CBL-Mutated JMML

*CBL*-mutated JMML accounts for approximately 10% to 17% of cases, making *CBL* the third most commonly mutated gene in this disorder after *PTPN11* and *NRAS* [4,109]. *CBL* encodes an E3 ubiquitin ligase that functions as a negative regulator of receptor tyrosine kinase signaling by targeting activated receptors for proteasomal degradation [4,68]. Mutations associated with JMML cluster within the linker region (affecting codon Y371) and RING finger domain, disrupting ubiquitin ligase activity while preserving the protein’s adaptor functions [32,109]. This paradoxically enhances downstream signaling through the PI3K-AKT and RAS-MAPK pathways, leading to elevated JAK2*-* and LYN*-*kinase expression and GM-CSF hypersensitivity—characteristic of JMML [32,109].

Like *NF1*, *CBL* functions as a tumor suppressor gene requiring bi-allelic inactivation for leukemogenesis, distinguishing it from the gain-of-function oncogenes *PTPN11*, *NRAS*, and *KRAS* [4]. A distinctive feature of *CBL*-mutated JMML is that approximately 85% of cases arise from germline *CBL* mutations with acquired UPD of chromosome 11q, rendering the mutant allele homozygous in hematopoietic cells [93]. The germline mutation represents the first hit, while somatic loss of heterozygosity through 11q-UPD constitutes the second hit that is positively selected in JMML cells [69,76]. This bi-allelic inactivation is required for full leukemic transformation, as the common p.Tyr371His-altered CBL protein induces cytokine-independent growth and constitutive phosphorylation of ERK, AKT, and S6 only when normal CBL expression is reduced [69]. Clinically, *CBL*-mutated JMML demonstrates remarkable heterogeneity, with many germline cases experiencing spontaneous resolution of myeloproliferative features without therapeutic intervention [11,92]. In a French cohort, 86% of the patients with *CBL*-mutated JMML had their disease managed with a watch-and-wait approach and they achieved long-term survival with partial or complete resolution of symptoms, although clonal hematopoiesis persisted in nearly all survivors [92]. However, approximately 15% of cases with *CBL*-mutated JMML harbor somatically acquired mutations and may follow a more aggressive clinical course [93]. Therefore, distinguishing germline from somatic *CBL* mutations through constitutional tissue testing is critical for prognostication and treatment planning, because neither clinical features nor methylation profiling reliably differentiate these two groups [93].

*CBL*-mutated cases are enriched in the LM subgroup, correlating with favorable outcomes [16,110,111] (see Section 10). Current guidelines recommend careful observation for most patients with germline *CBL*-mutated JMML, reserving HSCT for those with progressive disease regardless of mutation origin [4,92,93,97]. For patients who do undergo transplantation, stable mixed chimerism is frequently observed and does not indicate treatment failure [93,97,112].

**Table 4:** Molecular and clinical characteristics of JMML driver gene subtypes.

Feature	PTPN11	NRAS	KRAS	NF1	CBL
Frequency in JMML [4,16,94]	35–38%	15–20%	8–15%	10–15%	10–17%
Mutation origin [4,25,66,113]	Somatic (sporadic JMML) or germline (Noonan syndrome)	Predominantly somatic; rare germline RASopathy	Predominantly somatic; rare germline RASopathy	Germline (NF1 syndrome) requiring somatic second hit	Germline (~85%) or somatic (~15%); both require LOH
Mechanism of pathway activation [4,6,25]	Gain-of-function; disrupts SHP-2 autoinhibition	Gain-of-function; impairs GTPase activity	Gain-of-function; constitutively GTP-bound	Loss-of-function; bi-allelic inactivation eliminates GAP activity	Loss-of-function; bi-allelic inactivation impairs E3 ligase activity
Second-hit mechanism [11,66,110]	Not required (heterozygous)	Not required (heterozygous)	Not required (heterozygous)	17q-UPD (~40%), compound heterozygous (~36%), microdeletion + point mutation (~20%)	11q-UPD in ~85% of cases
Associated secondary mutations [66,98,111]	High frequency: *SETBP1*, *ASXL1*, *NF1*	Moderate: *SETBP1* co-occurrence reported	Lower mutational burden overall	Frequent: *ASXL1*, *SETBP1; FLT3* mutations contribute to progression	Exceedingly rare
Cytogenetic associations [16]	Variable	Variable	Enriched in monosomy 7	Variable	Rare cytogenetic abnormalities
Predominant methylation class [16,110]	HM	LM	IM	IM to HM	LM
Age at presentation [4,113]	Somatic: older (median ~3–4 years) Germline: infancy	Typically infants and toddlers	Variable	Older than other subtypes	Infancy to early childhood
Clinical course [4,11,66]	Aggressive; high relapse rate post-HSCT (~50%); elevated HbF	Heterogeneous; ranges from spontaneous resolution to aggressive disease	Heterogeneous; some respond durably to azacitidine alone	Rare somatic-only cases; 50% lack clinical NF1 stigmata	Somatic more aggressive than germline; requires closer monitoring
Overall prognosis [16,110]	Unfavorable	Favorable to intermediate	Intermediate	Unfavorable	Favorable (germline); intermediate (somatic)
Recommended management [4,11,97,113,114]	Prompt HSCT with pretransplant azacitidine; early immunosuppression withdrawal	Risk-stratified: observation for low-risk; HSCT for high-risk	Azacitidine may achieve HSCT-free remission; HSCT if progressive	HSCT mandatory; pretransplant azacitidine; high relapse risk	Observation for most germline cases; HSCT reserved for progression or somatic mutations

Abbreviations: GAP, GTPase-activating protein; HbF, fetal hemoglobin; HM, high methylation; HSCT, hematopoietic stem cell transplantation; IM, intermediate methylation; LM, low methylation; LOH, loss of heterozygosity; UPD, uniparental disomy.

## 6. JMML as a Bona Fide RASopathy

The designation of JMML as a bona fide RASopathy [5] rests on several converging lines of evidence: (1) more than 90% of cases harbor mutations in canonical RAS-MAPK pathway genes, a frequency of pathway-specific involvement unmatched by any other myeloid neoplasm [4,25]; (2) the same genes mutated somatically in sporadic JMML are mutated in the germline of RASopathy syndromes that predispose to JMML, demonstrating a direct biological continuum between developmental pathway dysregulation and malignant myeloproliferation [4,44]; (3) all five canonical driver mutations converge on a shared functional phenotype—GM-CSF hypersensitivity and myelomonocytic expansion—regardless of mutation origin or mechanism [29,30,32]; (4) the clinical spectrum of JMML recapitulates the variable expressivity characteristic of RASopathies, with germline cases sometimes undergoing spontaneous resolution while somatic mutations are more associated with aggressive disease [11,45,92]; and (5) both the ICC and WHO-5 classification recognize the RASopathy biology underlying JMML, albeit through different frameworks [18,20,21]. Collectively, these observations establish JMML not merely as a leukemia that co-occurs with RASopathies, but as a malignant manifestation of the same fundamental pathway dysregulation that defines the RASopathy spectrum [4,5].

## 7. Secondary Mutations

Beyond the five initiating canonical driver mutations, secondary genetic alterations occur in approximately 25% to 50% of JMML cases [25,33]. Whole-exome sequencing studies have identified recurrent mutations in *SETBP1* and *JAK3* as the most common secondary events [33]. Mutant *SETBP1* has been shown to enhance *NRAS*-driven MAPK-pathway activation and promote aggressive myeloid leukemia in murine models [115], while cooperation between *SETBP*1 and *ASXL1* mutations drives leukemic transformation through PP2A inhibition and HOXA pathway activation [116,117]. *SETBP1* mutations, affecting the SKI homologous domain, are detected in approximately 10% to 30% of patients when sensitive detection methods, such as droplet digital polymerase chain reaction, are used; these mutations are often subclonal at diagnosis and associated with markedly inferior event-free survival (18% vs. 51% for wild-type at 5 years) [33,118]. *JAK3* mutations similarly occur as secondary events and are implicated in disease progression rather than initiation [33].

Additional secondary mutations have been identified in genes encoding components of the PRC2, including *ASXL1* (5–10% of cases), splicing factors and transcriptional regulators [6,25]. The total number of somatic alterations present at diagnosis appears to be a major determinant of outcome, with higher mutational burden correlating with inferior survival [25]. Integration of secondary mutation status with DNA methylation profiling and clinical parameters has enabled the development of risk stratification models which identify patients at the highest risk of treatment failure who may benefit from novel therapeutic approaches [119].

## 8. Diagnosis

**Figure 2:**
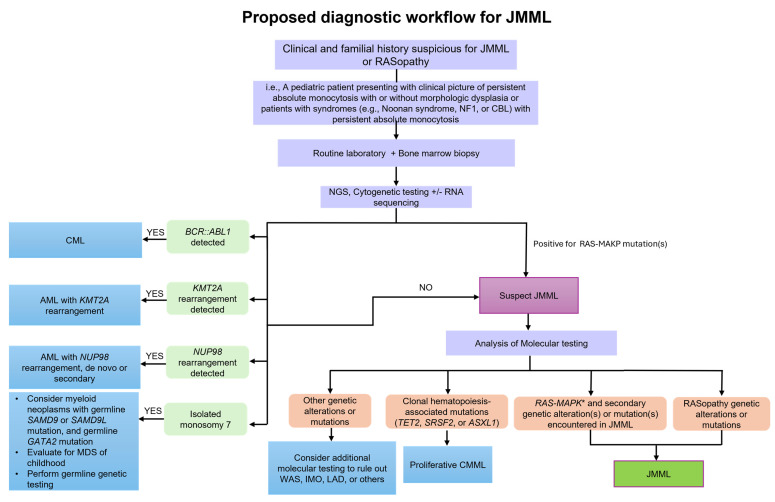
Proposed diagnostic workflow for JMML.*RAS-MAPK genetic alteration(s) or mutation(s) include *PTPN11*, *NRAS*, *KRAS*, *NF1*, and *CBL*. Abbreviations: JMML, juvenile myelomonocytic leukemia; CML, chronic myeloid leukemia; CMML, chronic myelomonocytic leukemia; IMO, infantile malignant osteopetrosis; LAD, leukocyte adhesion deficiency; MDS, myelodysplastic neoplasms; NGS, next-generation sequencing; WAS, Wiskott–Aldrich syndrome. This figure illustrates a proposed workflow which requires further clinical validation by larger-study cohorts.

The clinical and laboratory diagnostic criteria for JMML as defined by the WHO-5 and ICC are outlined in Table 5, with the WHO-4 included for comparison. To ensure an accurate diagnosis, a systematic diagnostic algorithm (Figure 2) should be followed that integrates (1) clinical features, (2) laboratory findings, (3) cytogenetic analysis, and (4) comprehensive molecular testing, while excluding benign and neoplastic mimickers.

## 9. Differential Diagnosis

With the widespread use of next-generation sequencing, molecular confirmation of JMML is now achievable in greater than 90% of cases. However, in 5% to 10% of cases lacking identifiable canonical RAS-pathway mutations, careful exclusion of alternative neoplastic and reactive entities with JMML-like features is essential. Because morphology alone is not specific, several myeloid neoplasms and reactive conditions must be considered in the differential diagnosis, particularly those cases presenting with monocytosis and myeloproliferative features in the pediatric population. Integration of patient age, clinical picture, morphologic findings, and molecular genetics is essential for accurate classification of JMML [4,11,18].

### 9.1. Juvenile Myelomonocytic Leukemia vs. Proliferative Chronic Myelomonocytic Leukemia

Chronic myelomonocytic leukemia (CMML) is the most important morphologic mimic of JMML, particularly proliferative CMML [120], sharing sustained monocytosis, splenomegaly, and myelomonocytic bone marrow expansion. The key distinguishing features are age and mutational profile. CMML occurs almost exclusively in older adults and is characterized by mutations in age-related clonal hematopoiesis genes (e.g., *TET2*, *SRSF2*, and *ASXL1*), which are largely absent in JMML [121,122,123,124]. Young-onset CMML (≤50 years) shows reduced *TET2/SRSF2* mutations and enriched *PTPN11* mutations, suggesting pathogenesis potentially more similar to JMML [120]. Peripheral blood monocyte subset repartitioning with classical monocyte expansion (>94%) can support a CMML diagnosis (Table 6) [121].

### 9.2. Juvenile Myelomonocytic Leukemia vs. Chronic Myeloid Leukemia

Chronic myeloid leukemia (CML) is a myeloproliferative neoplasm characterized by the *BCR::ABL1* rearrangement and by an incidence that increases with age [131]. Though rare, CML can occur in children, accounting for less than 3% of all pediatric leukemia cases [132]. Both CML and JMML are commonly present with splenomegaly, constitutional symptoms, and features of myeloproliferation, which may result in clinical diagnostic uncertainty. Peripheral monocytosis can also be seen as a variant of CML that often harbors a minor break point (p190) [133]. Therefore, testing for the *BCR::ABL1* rearrangement using fluorescence in situ hybridization (FISH), conventional karyotyping-banding, polymerase chain reaction (PCR), or other cytogenetic methods is essential to exclude CML according to JMML diagnostic guidelines.

### 9.3. Juvenile Myelomonocytic Leukemia vs. Juvenile Acute Myeloid Leukemia with *KMT2A* Rearrangement

Juvenile acute myeloid leukemia (AML) with *KMT2A* rearrangement (*KMT2A*-r) accounts for 15–20% of pediatric AML cases [134,135]. More than one hundred fusion partners have been identified, with *KMT2A::MLLT3* being the most common partner, and blasts often exhibit myelomonocytic differentiation [136]. This entity may overlap with JMML in clinical presentation, including splenomegaly, laboratory findings of monocytosis, low blast count, and elevated HbF levels [137]. According to WHO-5, blast count for *KMT2A*-r AML can be lower than 20%. Other fusion partners, such as *KMT2A::SEPT6* and *KMT2A::ELL*, have also been reported in cases initially presenting with features that mimic JMML [22]. Detecting 11q23/*KMT2A* rearrangement by cytogenetic or molecular methods plays a key role in establishing the diagnosis of *KMT2A*-r AML [22,137]. In contrast, the presence of RAS-pathway mutations may not reliably distinguish between *KMT2A-r* AML and JMML. *KMT2A-r* AML can also harbor *KRAS*, *NRAS*, or *PTPN11* mutations [138].

Juvenile myelomonocytic leukemia and *KMT2A-r* AML differ significantly in therapeutic approaches. Acute myeloid leukemia with *KMT2A*-r is typically treated with intensive chemotherapy regimens that include anthracyclines and high-dose cytarabine, whereas JMML may initially be managed with observation or low-dose chemotherapy; however, most patients require HSCT [113]. Given the importance of this distinction, the WHO-5 includes the absence of *KMT2A*-r as a required diagnostic criterion for JMML.

### 9.4. Juvenile Myelomonocytic Leukemia vs. Acute Myeloid Leukemia with *NUP98* Rearrangement

Acute myeloid leukemia with *NUP98* rearrangement (*NUP98-r*) is characterized by chromosomal alterations involving *NUP98* on chromosome 11p15.4, and has more than 20 reported fusion partner genes, including members of the *HOXA* gene cluster [139]. Additional somatic mutations in *FLT3-ITD* and *NRAS* have also been reported [139]. This entity is rare, accounting for approximately 3% to 4% of pediatric AML cases [140,141], but it is associated with an adverse prognosis [142]. Patients with AML harboring *NUP98*-r may initially present with clinical features resembling JMML, including leukocytosis and increased blast counts, often with myelomonocytic or monocytic differentiation [139]. Given the overlap in clinical and molecular features, RNA sequencing-based assays can detect cryptic fusion genes, such as *NUP98::NSD1* [139]. Identifying these fusions supports classification as AML according to the WHO-5 and ICC. Accurate detection of these rearrangements is therefore critical for resolving diagnostic uncertainty and guiding appropriate therapeutic management [139,143].

### 9.5. Juvenile Myelomonocytic Leukemia vs. Viral Infection-Induced Monocytosis (Reactive Monocytosis)

The nonspecific clinical presentation of JMML, including fever, skin rash, bleeding, and lymphadenopathy, along with complete blood count findings of leukocytosis, absolute monocytosis, thrombocytopenia, and left-shifted granulocytic maturation, may mimic viral infection [129,144,145]. Conversely, several viral infections including Epstein–Barr virus, human herpesvirus-6, cytomegalovirus, and parvovirus B19 can result in transient myeloproliferative and myelodysplastic changes in peripheral blood and bone marrow [145]. Selective hypersensitivity of myeloid progenitor cells to GM-CSF—a feature thought to play an important role in the pathogenesis of JMML—has also been observed in vitro in some cases of viral infections [129,146]. Among patients with such clinical presentations, one of the following may suggest an infectious etiology: (1) presence of viral cytopathic changes, (2) detection of viral nucleic acid by PCR, or (3) elevated virus-specific immunoglobulins M or G. However, JMML must be excluded through demonstrating RASopathy or excluding clonal cytogenetic or molecular abnormalities, as the clinical picture may be further complicated by the coexistence of JMML and a concurrent viral infection [144].

### 9.6. Juvenile Myelomonocytic Leukemia vs. RAS-Associated Autoimmune Leukoproliferative Disorder

RAS-associated autoimmune leukoproliferative disorder (RALD) is a nonmalignant autoimmune condition characterized by laboratory findings of monocytosis and somatic mutations in RAS-pathway genes including *KRAS* and *NRAS* [147]. These features significantly overlap with those observed in JMML, particularly in JMML cases with an indolent clinical course. While RALD is considered a chronic nonmalignant condition with an indolent clinical course, rare cases of progression to JMML or AML have been reported, suggesting potential for malignant transformation in some patients [147]. Studies have identified immunophenotypic differences in monocytes and granulocytes, as well as other peripheral blood findings that may distinguish RALD from JMML. These differences include (1) increased CD16 expression on monocytes (reflecting expansion of nonclassical monocytes), (2) increased CD14 expression on granulocytes, and (3) the presence of polyclonal CD10+ B-cell lymphocytosis [147]. In contrast, the presence of cytogenetic abnormalities, such as monosomy 7, favors a diagnosis of JMML over RALD and can serve as an important ancillary diagnostic finding [147].

### 9.7. Juvenile Myelomonocytic Leukemia vs. Wiskott–Aldrich Syndrome

Wiskott–Aldrich syndrome (WAS) is a rare X-linked recessive disorder caused by mutations in the *WASP* gene, which encodes the WAS protein (WASP) [148]. Studies suggest that WASP plays a vital role in the proliferation and differentiation of hematopoietic stem cells [149]. Patients typically present with eczema, recurrent infections, and microthrombocytopenia, along with laboratory findings that may include monocytosis and circulating immature myeloid and erythroid precursors [149,150]. Because some clinical and laboratory features overlap with those observed in JMML, molecular testing is essential to distinguish between these entities. Juvenile myelomonocytic leukemia is characteristically associated with mutations in genes involved in the RAS pathway or other noncanonical genetic mutations, whereas the diagnosis of WAS is confirmed by the presence of *WASP* gene mutations. Additionally, the presence of microthrombocytes favors a diagnosis of WAS and argues against JMML [149]. However, rare cases of WAS with normal or enlarged platelet size have been reported [151,152].

### 9.8. Juvenile Myelomonocytic Leukemia vs. Infantile Malignant Osteopetrosis

Infantile malignant osteopetrosis (IMO), also known as marble bone disease, is a rare autosomal recessive disorder characterized by osteoclast dysfunction, leading to decreased or absent bone resorption [153]. Patients may present with clinical and laboratory features that overlap with JMML, including hepatosplenomegaly, thrombocytopenia, anemia, monocytosis and circulating immature myeloid precursors [154]. However, several findings distinguish IMO from JMML. These include hypogammaglobulinemia, elevated alkaline phosphatase levels, and characteristic radiologic findings of diffusely dense and radiopaque bones, often demonstrating a bone-in-bone (endobone) appearance. Detection of mutations in *TCIRG1*, *CLCN7* and other related genes supports the diagnosis of IMO [153,154]. In addition, absence of clonal hematopoiesis further supports a diagnosis of IMO rather than JMML [154].

### 9.9. Juvenile Myelomonocytic Leukemia vs. Leukocyte Adhesion Deficiency

Leukocyte adhesion deficiency (LAD) is a rare primary immunodeficiency disorder characterized by defects in leukocyte adhesion and migration. Patients may present with clinical features that overlap with JMML, including recurrent infections, delayed separation of the umbilical cord, leukocytosis with neutrophilia, monocytosis, and hepatosplenomegaly [129]. However, the absence of mutations in the five canonical genes, which are commonly observed in JMML, can distinguish LAD from JMML. In addition, immunologic and molecular testing that confirms defects in leukocyte adhesion molecules and genes (*ITGB2*, *SLC35C1*, or *FERMT3*) further supports the diagnosis of LAD.

### 9.10. Juvenile Myelomonocytic Leukemia vs. Myeloid Neoplasms with Germline SAMD9 or SAMD9L Mutation and Germline GATA2 Mutation

*SAMD9* and *SAMD9L* genes are both located on chromosome 7 and have reported germline mutations [155]. Germline mutations of *SAMD9* have been reported to be associated with MIRAGE (myelodysplasia, infection, restriction of growth, adrenal hypoplasia, genital phenotypes, and enteropathy) syndrome [156]. Germline mutations of *SAMD9L* have been reported associated with ataxia-pancytopenia (ATXPC) syndrome [155,156,157]. Germline mutation of the *GATA2* gene located on chromosome 3q21.3 manifests as susceptibility to opportunistic infections, particularly *Mycobacterium avium* complex, lymphedema, pulmonary alveolar proteinosis, and cytopenia or monocytopenia with dysplasia [158], which differ from JMML. However, a subset of GATA2 deficiency patients with acquired *RAS* mutations could develop JMML-like features. Additionally, the above three conditions predispose affected individuals to bone marrow failure and pediatric MDS with risk of progression to AML, sometimes with acquired monosomy 7 [157,159,160]. Because monosomy 7 may also occur in JMML, the presence of monosomy 7 in the absence of a canonical RAS-pathway and noncanonical genetic mutations should prompt evaluation for underlying germline mutations in *SAMD9*, *SAMD9L*, or *GATA2* in children presenting with clinical features suggestive of JMML.

## 10. Prognosis and DNA Methylation-Based Risk Stratification

The clinical course of JMML varies widely, from spontaneous resolution to rapid progression and death. Traditional prognostic factors include older age at diagnosis, elevated HbF, low platelet count, and molecular subtype, with somatic *PTPN11* or *NF1* mutations associated with the poorest outcomes [6,7,96]. A recent French study of 119 transplanted patients identified four independent adverse prognostic factors: (1) age > 2 years; (2) time from diagnosis to HSCT ≥ 6 months; (3) monocyte count > 7.2 × 10^9^/L; and (4) presence of additional genetic alterations (*SETBP1*, *ASXL1*, *JAK3*, or additional RAS-pathway mutations) [119]. Patients with ≥3 predictors had a 5-year overall survival of only 34.2% compared with 100% for those with none [119]. However, genome-wide DNA methylation profiling has emerged as the most robust prognostic framework, consistently outperforming these conventional clinical and genetic risk factors [16,110,111].

Early studies focusing on selected candidate genes (including *BMP4*, *CALCA*, *CDKN2B*, and *RARB*) first demonstrated that increasing levels of promoter hypermethylation correlated with inferior outcomes, with a 5-year overall survival of approximately 6% for HM cases vs. 69% for LM cases [161]. Subsequent unbiased, genome-wide analyses confirmed these observations, revealing three reproducible methylation subgroups, HM, IM, and LM, which are strongly associated with distinct clinical outcomes [16,111]. Notably, patients in the LM subgroup cluster closer to healthy controls than to other JMML cases, and nearly all patients who experience spontaneous disease resolution belong to this favorable subgroup [111].

In 2021, an international consortium established a standardized methylation classification for JMML through pooled analysis of 255 patients, resulting in a highly accurate classifier applicable across multiple platforms, including EPIC arrays and MethylSeq [110]. This consensus confirmed that unfavorable prognostic parameters, including (1) somatic *PTPN11* mutations, (2) elevated HbF, (3) older age at diagnosis, and (4) secondary mutations, are significantly enriched in the HM subgroup, while the LM subgroup is associated with a favorable disease course [16,110]. Methylation subgroups correlate strongly with molecular subtypes: *PTPN11*-mutated cases cluster predominantly in the HM subgroup; *KRAS*-mutated cases cluster in the IM subgroup; and *CBL*- and *NRAS*-mutated cases cluster in the LM subgroup [16,110]. Monosomy 7 is the most common cytogenetic abnormality in JMML, occurring in approximately 25% of cases, and is enriched in the IM subgroup, particularly among KRAS-mutated cases [16]. Importantly, multivariable analysis identified DNA methylation subgroup as the sole independent predictor of overall survival, underscoring its dominant prognostic value [110]. The HM phenotype is associated with upregulation of the DNA methyltransferases *DNMT1* and *DNMT3B*, suggesting a biologic connection between RAS-pathway hyperactivation and aberrant epigenetic programming [16].

More recent work has streamlined methylation assessment using approaches based on NGS and machine learning-derived classifiers. These approaches can predict methylation classification from clinical parameters alone, facilitating broader clinical implementation [162,163]. As a result, DNA methylation profiling is now incorporated into standard JMML risk stratification in both Europe and the United States, and plays a central role in guiding risk-adapted therapeutic decisions, including identifying patients who may be managed with observation rather than immediate HSCT [12].

## 11. Current Treatment Approaches

The therapeutic landscape of JMML has evolved over the past decade. Although HSCT remains the only proven curative option for most JMML patients, contemporary outcomes show 5-year overall survival rates of approximately 65% to 75% and event-free survival rates of 50% to 65%, with post-transplant relapse persisting as the leading cause of treatment failure among 20% to 35% of the cases [11,119,164,165]. Advances in conditioning regimens have improved tolerability, with busulfan-based myeloablative protocols incorporating fludarabine and melphalan now favored; recent data suggests that cyclophosphamide-free approaches appear to reduce transplant-related toxicity without compromising efficacy [165,166]. The graft-versus-leukemia effect is central to achieving durable remissions for high-risk patients, and current recommendations emphasize early discontinuation of post-transplant immunosuppression to maximize this alloimmune response [11,97,119,165,167].

Hypomethylating agents, particularly azacitidine, have transformed pretransplant management. The AZA-JMML-001 trial demonstrated that upfront azacitidine monotherapy (75 mg/m^2^ administered daily for 7 consecutive days in a 28-day cycle) induced clinical partial responses in 61% of patients after three cycles, with 82% of transplanted patients remaining leukemia-free at median follow-up [114]. As a result, azacitidine is widely used as bridging therapy to cytoreduce disease burden before HSCT, particularly in patients with aggressive molecular subtypes such as *PTPN11*- or *NF1*-mutated JMML [114]. Notably, select patients with *KRAS*-mutated JMML have achieved sustained remissions with azacitidine alone without HSCT, suggesting potential for transplant-sparing approaches in this molecular subgroup [97,101].

A further paradigm shift has occurred with the recognition that a subset of patients, particularly those with germline *CBL* mutations, select *NRAS*-mutated cases with favorable clinical features or NS-MPD cases, may be managed with a watch-and-wait strategy [92]. In a recent French cohort, 35 (22% of the total cohort) patients were initially managed with observation alone; 86% of those achieved long-term survival with partial or complete resolution of myeloproliferative symptoms over a median follow-up of 6.5 years, although clonal hematopoiesis persisted in nearly all survivors [92]. Favorable predictors for successful watchful waiting in *NRAS*-mutated JMML cases include (1) age less than 30 months, (2) normal to slightly elevated HbF, (3) platelet count greater than 45 × 10^9^/L, (4) absence of secondary mutations, and (5) low DNA methylation profile [92].

Beyond these genotype-specific predictors, DNA methylation subgroup classification has been formally incorporated into clinical risk stratification as an independent prognostic tool, with patients in the low-methylation subgroup demonstrating the highest rates of spontaneous resolution across molecular subtypes [110,111]. Importantly, methylation classification provides prognostic information beyond genotype alone as multivariable analysis has identified it as the sole independent predictor of overall survival [110] (see Prognosis Section 10). This integrated genotype–epigenotype approach may spare up to 30% of JMML patients from HSCT-associated morbidity, though long-term monitoring remains essential given the persistence of clonal hematopoiesis and risk of late progression [92].

Emerging targeted therapies directed at aberrant RAS-MAPK signaling are now entering clinical evaluation. A Children’s Oncology Group phase II trial (ADVL1521) evaluating trametinib (MEK inhibitor) in relapsed/refractory JMML reported an objective response rate of 50%, with 7 of 10 patients completing maximum therapy or using trametinib as a bridge to HSCT and remaining alive at the median follow-up of 24 months [168,169]. Preclinical studies suggest synergistic activity when trametinib is combined with azacitidine in *PTPN11*-mutated disease [170]. *JAK* inhibitors have shown selective activity against *CBL*-mutated JMML cells in preclinical patient-derived induced pluripotent stem cell models [171], and PI3K/mTOR inhibitors demonstrate activity across multiple JMML subtypes [171]. Targeted agents specifically directed at secondary mutations such as *SETBP1* and *JAK3* remain an area of unmet need. Despite these advances, preventing post-transplant relapse remains a major challenge, underscoring the importance of developing effective maintenance approaches, including post-transplant hypomethylating therapy, donor-lymphocyte infusions, and emerging immunotherapeutic strategies [170,172]. Recent preclinical studies have identified promising immunotherapeutic approaches, including anti-CD52 (alemtuzumab) therapy disrupting disease propagation in JMML patient-derived xenograft models and ligand-based Chimeric Antigen Receptor (CAR) T cells targeting the GM-CSF receptor (CD116/CD131) demonstrating anti-proliferative effects against JMML CD34+ stem and progenitor cells [173,174].

## 12. Conclusions

### 12.1. JMML Is a Bona Fide RASopathy

More than 90% of cases harbor mutations in canonical RAS-pathway genes (*PTPN11*, *NRAS*, *KRAS*, *NF1*, and *CBL*), establishing JMML as the prototypical hematologic malignancy arising from aberrant RAS-MAPK signaling.

### 12.2. Classification Has Evolved

The WHO-5 reclassified JMML from MDS/MPN overlap to MPN, while the ICC introduced a *JMML-like* category for cases with emerging noncanonical drivers including *SH2B3/LNK* mutations and *ALK::ROS1* fusions.

### 12.3. Germline-Versus-Somatic Origin Determines Clinical Behavior

Approximately 35% to 45% of JMML cases arise among children with germline RASopathy mutations (*NF1*, *CBL*, and *PTPN11*), while 55% to 65% of JMML cases occur sporadically through acquired somatic mutations. This distinction profoundly influences prognosis and treatment decisions.

### 12.4. RASopathy Syndromes Confer Differential JMML Risk

In general, Noonan syndrome-associated myeloproliferation often resolves spontaneously. *NF1*-associated JMML behaves aggressively, requiring transplantation. CBL syndrome demonstrates heterogeneous outcomes with high rates of spontaneous resolution.

### 12.5. Level of DNA Methylation Predicts Prognosis

International consensus has established three methylation subgroups (high, intermediate, and low) that independently predict outcome and guide risk-adapted therapy, with LM cases enriched for spontaneous resolution.

### 12.6. Treatment Is Increasingly Guided by Integrated Genotype–Epigenotype Stratification

While allogeneic HSCT remains curative for most patients (5-year overall survival, 65–75%), DNA methylation profiling—now the sole independent predictor of overall survival—has been incorporated alongside genotype into risk-adapted treatment decisions. Watch-and-wait approaches are appropriate for up to 30% of patients with favorable molecular and epigenetic features. Azacitidine has transformed pretransplant management, and MEK inhibitors represent promising targeted therapy.

### 12.7. Emerging Drivers Expand the JMML Spectrum

Novel genetic alterations including *SH2B3/LNK* mutations and *ALK::ROS1* fusions define JMML-like disorders with distinct therapeutic implications, including potential responsiveness to JAK inhibitors and crizotinib.

## Figures and Tables

**Table 1:** Historical timeline of JMML classification.

Edition	Year	Category	Key Features
WHO, 3rd ed. [13]	2001	MDS/MPN overlap	First formal WHO classification; recognized as overlap syndrome
WHO, 4th ed. [14]	2008	MDS/MPN overlap	Maintained as overlap; refined diagnostic criteria
WHO, 4th ed., revised [15]	2016	MDS/MPN overlap	Continued as overlap; molecular criteria emphasized
WHO 5th ed. [18]	2022	MPN	Reclassified to MPN; RAS-pathway mutations required
ICC [20,21,21]	2022	Pediatric/germline predisposition disorders	Separate category; genetically defined; “JMML-like” created for emerging entities that would not meet the molecular criteria for JMML under either classification

Abbreviations: ICC, International Consensus Classification; JMML, juvenile myelomonocytic leukemia; MDS/MPN, myelodysplastic/myeloproliferative neoplasm; MPN, myeloproliferative neoplasm; WHO, World Health Organization Classification of Tumours.

**Table 2:** Genotype–phenotype correlations in PTPN11-associated myeloproliferation.

Mutation Location	Associated Phenotype	Clinical Implications	References
Codon Asp61 (exon 3)	NS with high JMML risk (~21%)	Close hematologic surveillance warranted	[45]
p.Thr73Ile (exon 3)	NS-MPD, milder course	Often self-resolving, watchful waiting may be appropriate	[45,46]
p.Glu76Lys (exon 3)	Severe neonatal NS (germline); sporadic JMML (somatic)	Rarely germline; aggressive disease when present	[46,48]
p.Asn308Asp/Ser (exon 8)	Typical NS, less cognitive impairment	Most common NS mutation; lower JMML risk	[48,49]
Other N-SH2 domain codons (71, 72; exon 3)	Elevated JMML risk	Subgroup requiring hematologic surveillance	[50]

Abbreviations: JMML, juvenile myelomonocytic leukemia; NS: Noonan syndrome; NS-MPD, Noonan syndrome-associated myeloproliferative disorder.

**Table 3:** JMML risk across different RASopathies.

RASopathy	Causative Gene(s)	Estimated JMML/MPD Risk	Clinical Course of JMML	Key Features
Noonan syndrome [45,46,47,55]	*PTPN11* (50%), *SOS1*, *RAF1*, *RIT1*, *KRAS*, *NRAS*, others	3–5.6% develop MPD features; ~10% of NS-MPD progresses to JMML	Generally benign; most cases resolve spontaneously; JMML is leading cause of death in *PTPN11*-NS	Codon Asp61 mutations: 21% develop MPD/JMML; p.Thr73Ile: milder course
Neurofibromatosis type 1 [4,66]	*NF1*	200- to 500-fold-increased risk vs. general population; ~10–15% of all JMML cases	More aggressive; requires HSCT in most cases	Bi-allelic *NF1* inactivation (LOH or compound heterozygous mutations) required
CBL syndrome [11,69,92,93]	*CBL*	~10–17% of all JMML cases have *CBL* mutations; majority are germline	Heterogeneous; many resolve spontaneously; some progress	Germline mutation + acquired 11q UPD; vasculitis may develop later
Costello syndrome [81]	*HRAS*	Rare; emerging association with JMML-like MPD	Limited data; recently recognized	~15% lifetime malignancy risk (mainly solid tumors); JMML-like MPD recently reported
Cardiofaciocutaneous syndrome [86,87,88,89]	*BRAF*, *MAP2K1*, *MAP2K2*, *KRAS*	Exceedingly rare; single case of transient MDS/MPN reported; monocytosis observed in ~32% of patients	Spontaneous resolution reported	Shared RAS-MAPK pathway dysregulation; germline *BRAF* c.721A>C associated with transient MDS/MPN
NRAS-associated RASopathy [90]	*NRAS*	Rare; case reports of MPD with Gly12 mutations	Variable	Germline Gly12/13 germline mutations associated with elevated MPD and tumor risk

Abbreviations: HSCT, hematopoietic stem cell transplantation; JMML, juvenile myelomonocytic leukemia; LOH, loss of heterozygosity; MPD, myeloproliferative disorder (used to denote transient, nonmalignant myeloproliferative processes associated with RASopathies, distinct from the WHO classification category of myeloproliferative neoplasm); NS-MPD, Noonan syndrome-associated myeloproliferative disorder; UPD, uniparental disomy.

**Table 5:** Comparison of diagnostic criteria of JMML between WHO-4, WHO-5 and ICC.

Criteria	WHO-4 [15]	WHO-5 [18]	ICC [20]
Classification category	MDS/MPN overlap	MPN	Pediatric and/or germline mutation-associated disorders
**Category 1: Clinical/hematologic**	**All 4 criteria mandatory**	**All criteria required**	**Criteria 1–2 present in most cases; 3–4 required**
Monocyte count	PB ≥ 1 × 10^9^/L	PB ≥ 1 × 10^9^/L	PB ≥ 1 × 10^9^/L
2. Blast percentage	<20% in PB and BM	<20% in PB and BM	<20% in PB and BM
3. Organomegaly	Splenomegaly	Organ infiltration (usually splenomegaly)	Splenomegaly
4. *BCR::ABL1*	Absent	Absent	Absent
5. *^a^ KMT2A*	Not specified	Absent (required)	Not specified
**Category 2: Genetic**	**≥1 required**	**≥1 required**	**≥1 required**
Somatic RAS-pathway mutations	*PTPN11*, *KRAS*, or *NRAS*	Clonal somatic *PTPN11*, *KRAS*, or *NRAS*	*PTPN11*, *KRAS*, *NRAS*, or ^b^*RRAS*
2. *NF1*	Clinical diagnosis or *NF1* mutation	Somatic/germline *NF1* with LOH or compound heterozygosity	Germline *NF1* with LOH or clinical diagnosis of NF1
3. *CBL*	Germline *CBL* with LOH	Somatic/germline *CBL* with LOH	Germline *CBL* with LOH
4. ^c^ Noncanonical drivers	Not specified	Fusions involving *ALK*, *PDGFRA*, *PDGFRB*, *ROS1*, *FLT3*	Not specified
**Category 3: Minor criteria**	**Required if no genetic criteria met**	**Required if no genetic ** **criteria met**	**≥2 required if no genetic criteria met**
	Monosomy 7 or other chromosomal abnormality, OR ≥ 2 of: HbF increased for age; myeloid/erythroid precursors in PB; GM-CSF hypersensitivity; pSTAT5 hyperphosphorylation	Monosomy 7 or other chromosomal abnormality, OR ≥ 2 of: HbF increased for age; myeloid/erythroid precursors in PB; GM-CSF hypersensitivity; pSTAT5 hyperphosphorylation	Circulating myeloid/erythroid precursors; HbF increased for age; thrombocytopenia with hypercellular marrow; GM-CSF hypersensitivity
**Diagnostic exclusions**	Other MDS/MPN (e.g., CMML)	*BCR::ABL1* fusion; *KMT2A* rearrangement; ^d^ isolated monosomy 7 without RAS-pathway mutation	*BCR::ABL1* fusion; *KMT2A* rearrangement; ^e^ viral infections; infantile malignant osteopetrosis; Wiskott–Aldrich syndrome; *GATA2/SAMD9/SAMD9L*-associated disorders

Abbreviations: BM, bone marrow; CMML, chronic myelomonocytic leukemia; GM-CSF, granulocyte–macrophage colony-stimulating factor; HbF, fetal hemoglobin; ICC, *International Consensus Classification*; LOH, loss of heterozygosity; MDS/MPN, myelodysplastic syndrome/myeloproliferative neoplasm; MPN, myeloproliferative neoplasm; PB, peripheral blood; pSTAT5, phosphorylated signal transducer and activator of transcription 5; WHO, World Health Organization. ^a^ *KMT2A* rearrangement exclusion is explicitly required by WHO-5 to distinguish JMML from infant acute leukemia with monocytic features [18]. ^b^ The ICC uniquely includes RRAS among canonical driver genes, in addition to *PTPN11*, *KRAS*, and *NRAS* [20]. ^c^ Noncanonical drivers recognized by WHO-5 include activating fusions involving receptor tyrosine kinases (*ALK*, *PDGFRA*, *PDGFRB*, *ROS1*, and *FLT3*) that signal upstream of RAS; these are particularly reported in older patients with JMML [18]. ^d^ WHO-5 explicitly excludes cases with isolated monosomy 7 without an identifiable RAS-pathway mutation from the JMML category. ^e^ ICC diagnostic exclusions include viral infections (CMV, EBV, and HHV-6), infantile malignant osteopetrosis, Wiskott–Aldrich syndrome, and germline predisposition syndromes (GATA2-, SAMD9-, and SAMD9L-associated disorders) that may present with JMML-like features and monosomy 7 [20].

**Table 6:** Distinct features between JMML, proliferative CMML, and CMML in the young.

Feature	JMML	pCMML	CMML in the Young
Disease category	WHO-5: Myeloproliferative neoplasm [18] ICC: Pediatric and/or germline mutation-associated disorder [21]	MDS/MPN	MDS/MPN
Age at onset	Early childhood; median age ~1.8 years [4,5,18]	Older adults; median age ~71–74 years [123,125]	≤50 years (rare; ~20% of CMML cases) [120]
Epidemiology	Rare, pediatric leukemia	Rare, myeloid neoplasm of aging	Rare; distinct clinical entity [120]
Key biologic driver	Primary RAS-MAPK pathway activation (bona fide RASopathy) [4,5,18]	Age-related clonal hematopoiesis with secondary RAS-pathway activation [5,123,126]	Distinct pathogenesis; less reliance on age-related CH mutations [120]
RAS-pathway mutations	Present in >90–95% of cases (*PTPN11*, *NRAS*, *KRAS*, *NF1*, *CBL*) [4,5,18]	Present in ~30% overall; enriched in proliferative subtype [123,127]; *NRAS* (15%), *KRAS* (10%), *CBL* (15%), and *PTPN11* (5%) [122]	*PTPN11* enriched (13% vs. 3% in o-CMML; *p* = 0.034) [120]
CH mutations	Typically absent [5,126]	Present in 80–90% (*TET2* ~60%, *SRSF2* ~50%, *ASXL1* ~40%) [5,123]	Significantly reduced: *TET2* (6% vs. 57%), *SRSF2* (16% vs. 46%) [120]
Germline predisposition	~30% (NF1, CBL syndrome; some PTPN11) [4,5,18]	Rare	Rare
Prognosis	Variable; some spontaneous regression in germline types (~15%) [4,5,18]	Poor; median survival < 36 months [128]	Similar OS and LFS to o-CMML despite molecular differences [120]
Leukemic transformation risk	Variable [4,5,18]	~15–30% [128]	Similar to CMML [120]
Therapeutic response	Limited response to HMAs or hydroxyurea [129]	HMAs and hydroxyurea are commonly used for cytoreduction [130]	Similar to oligomonocytic CMML; HSCT is only curative option [120]
Key diagnostic distinction	Pediatric age + isolated RAS-pathway mutations [4,5,18]	Adult age + CH mutations ± *RAS* mutations	Young adult (≤50 years) + reduced CH mutations + enriched *PTPN11* + prominent organomegaly [120]

Abbreviations: CH, clonal hematopoiesis; CMML, chronic myelomonocytic leukemia; HMA, hypomethylating agent; HSCT, hematopoietic stem cell transplantation; ICC, *International Consensus Classification*; LFS, leukemia-free survival; MDS/MPN, myelodysplastic/myeloproliferative neoplasm; MPN, myeloproliferative neoplasm; OS, overall survival; pCMML, proliferative CMML; WHO, World Health Organization.

## Data Availability

No new data were created or analyzed in this study.
